# The Prognosis of Adult Burkitt’s Cell Leukemia in Real-Life Clinical Practice

**DOI:** 10.4274/tjh.2015.0088

**Published:** 2016-12-01

**Authors:** Ümit Yavuz Malkan, Gürsel Güneş, Hakan Göker, İbrahim C. Haznedaroğlu, Kadir Acar, Eylem Eliaçık, Sezgin Etgül, Tuncay Aslan, Seda Balaban, Haluk Demiroğlu, Osman İ. Özcebe, Nilgün Sayınalp, Salih Aksu, Yahya Büyükaşık

**Affiliations:** 1 Hacettepe University Faculty of Medicine, Department of Hematology, Ankara, Turkey; 2 Gazi University Faculty of Medicine, Department of Hematology, Ankara, Turkey

**Keywords:** Burkitt’s cell leukemia, prognosis

## Abstract

**Objective:**

Many studies reported an improved prognosis in patients with Burkitt’s lymphoma obviating the need of stem cell transplantation. However, prognosis of the advanced disease [i.e. Burkitt’s cell leukemia (BCL)] has not been reported with current treatment modalities except for a few prospective trials. The aim of this study is to compare the prognoses of BCL patients with similarly treated and nontransplanted patients with other types of acute lymphoblastic leukemia (ALL) and with ALL patients that underwent allogeneic stem cell transplantation (ASCT) in their first remissions.

**Materials and Methods:**

In this retrospective analysis, BCL patients aged between 16 and 63 who were admitted between 2000 and 2014 to the hospitals of Hacettepe or Gazi University and were treated with intensive therapies aimed at cure were included. All ALL patients who were treated with a similar protocol not including transplantation during the same period (NT-ALL group) and all ALL patients who underwent ASCT in the first complete remission during the same period (T-ALL group) served as control groups.

**Results:**

The central nervous system or extramedullary involvement rates, lactate dehydrogenase levels, and white blood cell counts at diagnosis were higher in the BCL group than the NT-ALL group and these differences were significant. BCL patients had disease-free survival (DFS) durations comparable with the T-ALL cohort but NT-ALL patients had significantly shorter DFS durations. Both cumulative relapse incidence and cumulative nonrelapse mortality were higher in NT-ALL patients compared to the T-ALL group and BCL patients.

**Conclusion:**

DFS in BCL patients treated with a widely accepted modern regimen, R-HyperCVAD, is comparable to results in other ALL patients receiving allogeneic transplantation. Our results are in agreement with a few prospective noncomparative studies suggesting no further need for stem cell transplantation in BCL.

## Abstract (tr)

**Amaç:**

Yapılan birçok çalışmada Burkitt lenfomanın seyrinin düzeldiği, hatta kemik iliği nakli ihtiyacının ortadan kalktığı ileri sürülmektedir. Ancak birkaç ileriye dönük çalışma haricinde, güncel tedavi yöntemleri altında hastalığın lösemik formunun seyri hakkında araştırma yapılmamıştır. Bu çalışmanın amacı Burkitt hücreli lösemi (BHL) hastalarının klinik seyrinin, benzer tedavi alan ve transplantasyon uygulanmayan diğer akut lenfoblastik lösemi (ALL) hastaları ve ilk remisyonlarında allojenik kök hücre nakli (AKHN) uygulanan ALL hastalarıyla kıyaslanmasıdır.

**Gereç ve Yöntemler:**

Geriye dönük olarak tasarlanan bu çalışmaya yaşları 16 ile 63 arasında değişen, 2000 ile 2014 yılları arasında Hacettepe ve Gazi Üniversitesi Hastaneleri’ne başvurup kür amacıyla intensif tedavi verilen BHL hastaları alınmıştır. Transplantasyon haricinde benzer tedavi protokolüyle tedavi edilen tüm ALL hastaları (NT-ALL) ve aynı dönemde ilk tam remisyonlarında AKHN uygulanan hastalar (T-ALL) kontrol grupları olarak çalışmaya alınmışlardır.

**Bulgular:**

Santral sinir sistemi ya da ekstra medüller tutulum hızları, tanı anındaki laktat dehidrogenaz düzeyleri ve beyaz küre sayısı BHL hastalarında NT-ALL hastalarına göre istatistiksel olarak anlamlı olacak şekilde daha yüksekti. BHL hastaları T-ALL hastalarıyla benzer hastalıksız sağkalım (HS) süresine sahip olmakla beraber, NT-ALL hastalarında HS süresi önemli oranda azalmıştı. Kümülatif nüks insidansı ve kümülatif nüks dışı ölümler NT-ALL hastalarında T-ALL ve BHL hastalarında kıyasla daha fazlaydı.

**Sonuç:**

Sonuç olarak, geniş kabul gören modern bir rejim olan R-HyperCVAD ile tedavi edilen BHL hastalarında HS süresi allojenik transplantasyon uygulanmış diğer ALL hastaları ile benzer bulundu. Bizim çalışmamızın sonuçları, literatürde ileri dönük dizayn edilmiş ancak kontrol grupları ile karşılaştırma olmadan yapılmış ve BHL’de transplantasyon gerekmediğini öne süren az sayıdaki çalışma ile örtüşmektedir.

## INTRODUCTION

In the last decade, many studies reported an improved prognosis in patients with Burkitt’s lymphoma obviating the need for stem cell transplantation. There is a general consensus that the prognosis of Burkitt’s lymphoma is closely related to the disease stage and degree regarding the involvement of bone marrow and peripheral blood. However, prognosis of the advanced disease (i.e. Burkitt’s cell leukemia) specifically has not been reported with current treatment modalities except for a few prospective trials, which may not reflect everyday real-life clinical practices with their own limitations.

The aim of this study is to compare the prognoses of Burkitt’s cell leukemia patients with similarly treated and nontransplanted patients with other types of acute lymphoblastic leukemia and with acute lymphoblastic leukemia patients that underwent allogeneic stem cell transplantation in their first remissions.

## MATERIALS AND METHODS

### Study Population

In this retrospective analysis, Burkitt’s cell leukemia patients aged between 16 and 63 years who were admitted between 2000 and 2014 to the hospitals of Hacettepe or Gazi University and treated with intensive therapies aimed at cure were included in the study. Twenty-five patients who were treated with HyperCVAD ± rituximab were included in the study; as only one patient was treated with the R-EPOCH regimen, that patient was excluded from the study. The diagnosis of Burkitt’s cell leukemia was made based on the presence of characteristic morphological (FAB L3 morphology and >95% Ki-67 proliferation index) or cytogenetic/molecular (specific translocations involving MYC at band 8q24 or MYC rearrangement in fluorescence in situ hybridization analysis) properties and mature B-cell immunophenotype (TdT negativity plus sIg positivity of >20% or κ/λ light-chain clonality). The minimal criterion for the diagnosis of a leukemic disease condition was more than 25% bone marrow involvement. All acute lymphoblastic leukemia patients who were treated with a similar protocol not including transplantation during the same period (NT-ALL group) and all acute lymphoblastic leukemia patients who underwent allogeneic stem cell transplantation in the first complete remission during the same period (T-ALL group) served as control groups.

### Treatment Protocols

Specifics of the HyperCVAD ± rituximab regimen, including central nervous system (CNS) prophylaxis and treatment strategies, were as described by Thomas et al. [1]. Chemotherapy consisted of 8 alternating courses without maintenance therapy. When given, rituximab was administered during courses 1 to 4. Odd courses (1, 3, 5, 7) were HyperCVAD. When given, rituximab was administered at 375 mg/m2 i.v. over 2 to 6 h on days 1 and 11 of HyperCVAD and on days 2 and 8 of MTX and ara-C, during the first 4 courses.

### Study End-Points and Statistical Analysis

Numerical descriptive data were expressed as median (minimum-maximum). Continuous and categorical data were compared with the t-test and chi-square test, respectively. Primary endpoints of the study were complete remission (CR) rate, disease-free survival (DFS), and overall survival (OS). OS was calculated from diagnosis to the date of mortality of any reason. DFS was analyzed in CR patients from date of CR attainment to relapse or death in remission. The patients who did not die and those who did not relapse or die in remission at last follow-up were censored at this time for OS and DFS computations, respectively. Cumulative relapse (CRI) and cumulative nonrelapse mortality incidences (CNRMI) were computed for patients who attained CR, from the date of CR until relapse or nonrelapse mortality (NRM), respectively. The patients who did not relapse or die in remission at last follow-up were censored at this time. Relapse was considered a competing risk for NRM, and NRM was considered a competing risk for relapse during CRI and CNRMI computations. Categorical and continuous data were compared by the chi-square and independent-samples t-test, respectively. Survival analyses were computed by the Kaplan-Meier method. Comparisons of survival rates were done by the log-rank test. CRI and CNRMI were calculated according to Gray’s test [2] as described by Scrucca et al. [3]. Cumulative incidences were calculated by means of the statistical software environment R, Version 2.15.2 (The R Foundation for Statistical Computing, Vienna, Austria) [4]. SPSS 17.0 (SPSS Inc., Chicago, IL, USA) was used for other statistical analyses.

## RESULTS

**Figure 1:**
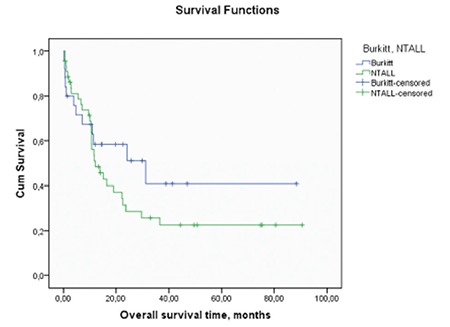
Overall survival time for Burkitt and NTxALL groups.

**Figure 2:**
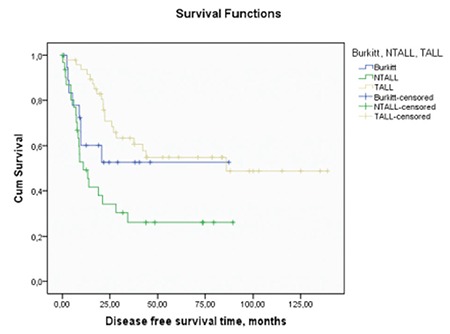
Disease-free survival time of Burkitt, NTxALL, and TxALLgroups.

**Figure 3:**
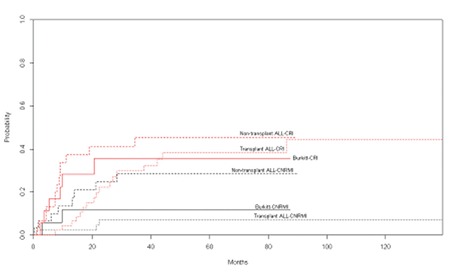
Cumulative relapse and cumulative nonrelapse mortalityincidences of Burkitt, NTxALL, and TxALL groups.

T-ALL patients were frequently referred after remission attainment from other centers. Some of these patients’ baseline parameters were missing. There were 25, 44, and 48 patients in the Burkitt’s cell leukemia, NT-ALL, and T-ALL groups, respectively. Important baseline characteristics of Burkitt’s cell leukemia and NT-ALL patients are presented in Table 1. All 25 Burkitt’s cell leukemia patients had been treated with the HyperCVAD ± rituximab regimen and were not transplanted. Rituximab treatments were given to most of the Burkitt’s cell leukemia patients; only 3 Burkitt’s cell leukemia patients had not received rituximab. Only nontransplanted acute lymphoblastic leukemia (NT-ALL) patients who were treated with HyperCVAD were selected as controls. Median numbers of HyperCVAD ± rituximab regimens given to Burkitt’s and NT-ALL patients were 8 and 7.5, respectively. The CNS or extramedullary involvement rate, lactate dehydrogenase levels, and white blood cell count at diagnosis were higher in the Burkitt’s group than the NT-ALL group and these differences were significant (p=0.008, p=0.016, and p=0.036, respectively). We also analyzed the chemotherapy intervals between treatment cycles. There was no significant difference between the intervals of treatment cycles for the Burkitt’s cell leukemia and NT-ALL groups. The median (95% confidence interval) OS time for all 25 Burkitt’s cell leukemia patients was 31.1 (3.1-59.1) months. The mean (95% confidence interval) DFS time for Burkitt’s cell leukemia patients was 50.0 (30.9-69.2) months (median not reached). After analyzing the prognosis, we further analyzed the induction chemotherapy results and OS in the patients with Burkitt’s cell leukemia receiving HyperCVAD and similarly treated nontransplanted acute lymphoblastic leukemia patients. Transplanted acute lymphoblastic leukemia patients were preferentially not included in this analysis because the majority of them had been referred after remission attainment from other centers. After the induction therapy, 5 patients died, 19 patients achieved CR, and 1 patient had no response in the Burkitt’s cell leukemia group. Four patients died, 33 patients achieved CR, and 7 patients had no response in the NT-ALL group. We achieved a 76% CR rate in the Burkitt’s group and a 75% CR rate in the NT-ALL group (p=0.182). The median (95% confidence interval) OS time for the Burkitt’s and NT-ALL groups were 31.1 (3.1-59.1) and 12.1 (7.0-17.3) months, respectively (p=0.261). There was no significant difference between the two groups (Figure 1). After obtaining these results, we analyzed the DFS, CRI, and CNRMI in the 3 groups. The mean DFS time for the Burkitt’s, NT-ALL, and T-ALL groups was 50.0±9.7, 31.4±6.7, and 83.3±9.1 months, respectively (p=0.002). There was a significant statistical difference between these 3 groups (Figure 2). Burkitt’s cell leukemia patients had DFS durations comparable with the T-ALL cohort (50.0±9.7 vs. 83.3±9.1 months, respectively; p=0.17), but NT-ALL patients had significantly inferior DFS durations compared to the T-ALL group (31.4±6.7 vs. 83.3±9.1 months, respectively; p=0.001). Both CRI (45.4% [standard error, SE: 9.8%], 38.2% [SE: 7.8%], and 35.7% [SE: 12.5%] at the 80th month; p=0.04) and CNRMI (28.5% [SE: 8.8%], 6.8% [SE: 3.9%], and 11.5% [SE: 8%] at the 80^th^ month; p=0.03) were higher in NT-ALL patients compared to the T-ALL group and Burkitt’s cell leukemia patients (Figure 3).

## DISCUSSION

As stem cell transplantation for Burkitt’s cell leukemia has been abandoned in the modern era, we preferred to evaluate success of current treatment in these cases by comparing them with similarly treated NT-ALL and T-ALL patients. Currently, allogeneic stem cell transplantation is deemed necessary in adult acute lymphoblastic leukemia during the first complete remission. We thought that in the absence of possibilities of evaluating the value of allogeneic stem cell transplantation in Burkitt’s cell leukemia by a randomized study or by using a currently transplanted Burkitt’s cohort, the necessity of treatment could be weighed by comparison of Burkitt’s cell leukemia cases with T-ALL and NT-ALL patients. Transplanted acute lymphoblastic leukemia patients had the best DFS, significantly better than that of nontransplanted patients. However, no DFS advantage could be observed in transplanted patients compared to Burkitt’s cell leukemia patients.

In our study, we achieved a 76% CR rate after induction therapy in Burkitt’s cell leukemia cases. In a study conducted by a German group, an 86% CR rate was achieved in Burkitt’s cell leukemia patients [5]. In another study conducted in Italy, investigators obtained a 79% CR, 8% no-response rate, and 13% death rate in Burkitt’s lymphoma and leukemia patients after induction chemotherapy [6]. In our study, we obtained 76% CR, 20% death, and 4% no-response rates in Burkitt’s cell leukemia patients. The induction death rate in our study was higher than that of the Italian study. The reason for this difference may be that participants were in an advanced stage of disease (Burkitt’s cell leukemia) in our study, whereas patients in the Italian study had both Burkitt’s lymphoma and leukemia. Furthermore, in the Italian study, investigators found a relapse rate of only 7% in patients treated with an intercycle interval of ≤25 days. We found the CRI of Burkitt’s cell leukemia patients as 35.7%, which was much higher. The intercycle interval could be the reason for this difference, because in our study the mean duration of all chemotherapy intercycles was longer than 25 days. It is known that men are more commonly affected by Burkitt’s disease with a 3-4:1 ratio [7]. Similarly, in our cohort, men were more common, with a ratio of 2.5:1.

In reported clinical trials, the prognosis for Burkitt’s lymphoma is generally favorable, with median survivals of 75%-90% with modern chemoimmunotherapy regimens [1,8]. An analysis of the Surveillance Epidemiology and End Results (SEER) database was less encouraging, however, with a 5-year OS rate of 56% and better survival seen in younger patients with lower-risk disease (87% and 71% for patients aged 0-19 years and for patients with low-risk disease, respectively) [9,10]. The impact of age on outcomes is likely multifactorial and reflects increased treatment toxicity or decreased treatment intensity in older individuals, as well as the potential misclassification of disease in this population. In our study the mean OS time for all 25 Burkitt’s cell leukemia patients was 43.6±9.2 months. Burkitt’s lymphoma principally involves the lymph nodes, bone marrow, and CNS, but it may also present with peripheral blood involvement [11]. In our study, peripheral blood involvement was present in 66% of cases. A limitation of our study is that in the T-ALL group DFS duration after first CR was found comparable but OS duration was not calculable.

In conclusion, DFS in Burkitt’s cell leukemia patients treated with a widely accepted modern regimen, R-HyperCVAD, is comparable to that of allogeneic transplanted patients of acute lymphoblastic leukemia. Although this study has some disadvantages inherent to its retrospective design, use of non-Burkitt’s control groups, and a limited patient numbers, we think that a better comparative study design is practically impossible due to the absence of a large transplanted Burkitt’s cohort and ethical issues in planning a prospective study including transplantation in these patients. Our results are in agreement with the few prospective noncomparative studies [12,13], suggesting no further need for stem cell transplantation in Burkitt’s cell leukemia.

## Ethics

Informed Consent was taken during the hospital admission of the patients, additional Ethics Committee Approval was not applicable based on the nature of this retrospective analysis.

## Figures and Tables

**Table 1:**
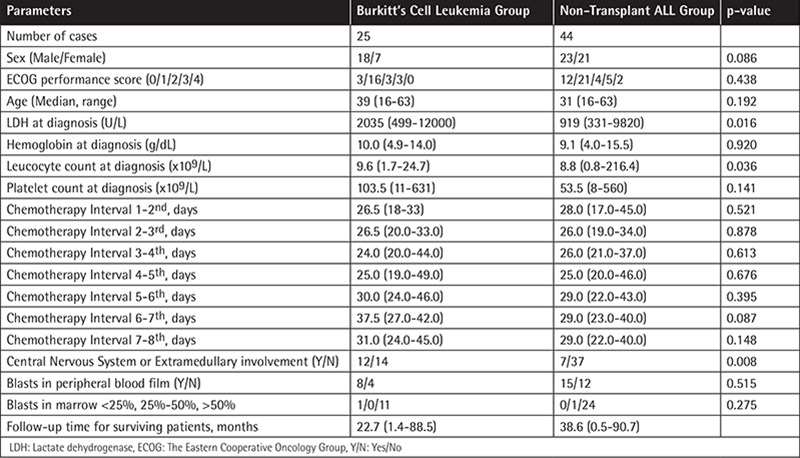
Main baseline characteristics and follow-up durations of Burkitt’s cell leukemia and similarly treated non-transplantacute lymphoblastic leukemia patients.
